# Synthesis, Physical Properties and Electrocatalytic Performance of Nickel Phosphides for Hydrogen Evolution Reaction of Water Electrolysis

**DOI:** 10.3390/nano12172935

**Published:** 2022-08-25

**Authors:** Gaoyang Liu, Faguo Hou, Shanlong Peng, Xindong Wang, Baizeng Fang

**Affiliations:** 1 Department of Energy Storage Science and Technology, University of Science and Technology Beijing, 30 College Road, Beijing 100083, China; 2 Department of Metallurgical and Ecological Engineering, University of Science and Technology Beijing, 30 College Road, Beijing 100083, China; 3 Department of Chemical and Biological Engineering, University of British Columbia, 2360 East Mall, Vancouver, BC V6T 1Z3, Canada

**Keywords:** nickel phosphide, amorphous, crystalline, hydrogen evolution reaction, water electrolysis

## Abstract

Nickel phosphides have been investigated as an alternative to noble metals and have emerged as potential catalysts that can efficiently catalyze the hydrogen evolution reaction (HER). However, the impacts of facet morphology and crystal structure of the nickel phosphides on their catalytic reactivity have not been systematically investigated. Herein, nickel phosphides with different crystalline states were prepared through a facile calcination treatment. It was found that the calcination treatment had important effects on the phase compositions, morphologies, and crystallinity of nickel phosphides, which are closely related to their HER activity. Generally, the crystallized Ni-P catalysts exhibited faster kinetics than the amorphous Ni-P. In particular, the Ni-P 300 showed remarkable HER performance with $\eta_{10}$ of ca. 65 mV, along with a very low Tafel slope of ca. 44 mV dec^−1^ due to the increased catalytically active sites. Furthermore, the Ni-P 300 exhibited negligible decay during the 140 h galvanostatic electrolysis, showing better catalytic stability than the commercial Pt/C catalyst. Compared with the amorphous Ni-P, the boosted HER activity of the Ni-P 300 could benefit from the mixed nanocrystalline Ni_2_P and Ni_3_P, which could contribute to the H_ads_ adsorption/desorption abilities and helped provide more activity sites, promoting the HER performance.

## 1. Introduction

Hydrogen has been strongly proposed as a replacement for traditional fossil fuels in a variety of applications [1,2,3,4,5,6,7,8]. Therefore, it is necessary to develop large-scale environmentally friendly and economical technologies for hydrogen production [9,10,11,12]. Among the various options, proton exchange membrane (PEM) water electrolysis (WE) has been considered as one of the most promising technologies for hydrogen production, mainly because of its high efficiency, high purity, good safety and reliability [13].

Generally, noble metals, e.g., Pt and Pd, are ideal catalysts for the hydrogen evolution reaction (HER) [14,15]. However, the high price of Pt and Pd could be a limiting factor for large-scale deployment of PEMWE technology. Development of non-precious metal electrocatalysts for the HER is, thus, of great importance and will help reduce the cost of PEM water electrolyzers [16,17]. Transition metal phosphides, sulfides, carbides, and other non-precious metals are potential non-precious catalysts for the HER and have demonstrated good corrosion resistance and high electrical conductivity [18,19,20]. In these compounds, the coordination of chemical bonds between P, S or C with transition metal (Ni, Co, Fe, etc.) atoms and the “group effect” significantly improve the hydrogen adsorption and desorption capacity on the surface of the compound, thereby accelerating the reaction rate of the intermediate step during the HER. So far, various transition metal compounds, e.g., Ni [21], Co [22], Fe [23], and Mo [24,25]-based materials have been successfully examined as HER catalysts for efficient water electrolysis. Among them, nickel phosphides have been investigated as a replacement for noble metal catalysts and showed excellent catalytic activity towards the HER, which remains an important research topic [26]. 

In nickel phosphides, the P atom is most likely located where hydrogen adsorption occurs and this is primarily due to its electronegativity. The adsorbed active hydrogen (H_ads_) on P tends to desorb H_2_. Due to the “coordination effect” of the Ni-P bond, it helps reduce the adsorption capacity of Ni atoms, promote desorption of H_ads_ and restore Ni vacancy [27]. Thus, the arrangement and coordination of the surface P and Ni atoms, which will affect the rate of the intermediate step in the HER, influence the catalytic reactivity and selectivity of nickel phosphides catalysts [21,28]. Various factors can affect the composition, the crystal structure as well as the exposed crystal facets of nickel phosphide catalysts. Reducing the grain size of the nickel phosphides can increase the number of active coordinated P and Ni catalytic sites, and simultaneously modify the electronic properties [29,30]. The crystal size and structure can be tuned due to different atomic ratios of P and Ni atoms, therefore resulting in different arrangements and coordination of the surface atoms [27,31]. For nickel phosphides with a high phosphorus content, the excess P atoms stabilize on the surface of crystalline nickel phosphide via dangling bonds to threefold Ni atoms. The growth of an ordered crystal structure of nickel phosphide nanocrystals is blocked, and an amorphous nickel phosphide structure is, thus, formed. In amorphous nickel phosphide, a large number of coordinated P atoms and Ni atoms are exposed, which constitute active sites for the HER. For nickel phosphides with a low phosphorus content, nickel phosphide nanocrystals can be developed along one crystal facet, and form long-range ordered crystalline materials, such as crystalline Ni_2_P [32]. Research has shown that the exposed (001) facets of crystalline Ni_2_P contribute significantly to the high catalytic activity of the Ni_2_P nanoparticles [33]. Recently, great efforts have been devoted to understanding the role of the facet morphology and crystal structure in the catalytic reactivity of nickel phosphides. Increasing the number of coordinated P and Ni atoms exposed on different crystal plane boundaries of tiny crystalline nickel phosphides may improve the active sites of HER [26,34].

Herein, to increase the activity of nickel phosphides, we attempted to tune the composition, the crystal structure, as well as the exposed crystal facets of nickel phosphides via a facile method. This was initiated by first preparing highly dispersible amorphous nickel phosphides. Increasing the overall specific surface area of the amorphous nickel phosphides leads to enhanced activity of the catalysts for the HER. Next, the obtained amorphous nickel phosphides were calcined, and nickel phosphides with different crystalline states were obtained. The morphology, crystal structure and the exposed crystal facets of the as-calcined materials were characterized by various surface techniques. Furthermore, the electrocatalytic reactivity and selectivity of the different catalysts were further investigated in order to reveal the structure–activity relationship.

## 2. Experimental 

### 2.1. Materials 

All chemical reagents were purchased from Sinopharm Chemical Reagent Beijing Co., Ltd., Beijing, China. Commercial 20 wt% Pt/C (Johnson Matthey Co., London, UK) and all other chemicals were used as received without any further purification. 

### 2.2. Synthesis of Amorphous Ni-P 

A facile and modified electroless plating technique was employed for the synthesis of amorphous nickel phosphides [35]. First, a mixed salt solution (150 mL) containing 20 g of NaH_2_PO_2_·H_2_O, 22.5 g of NiCl_2_·6H_2_O and an appropriate amount of sodium acetate were prepared. Then, the pH value of this solution was adjusted to 3 by addition of 0.1 M sodium hydroxide (NaOH). The electroless plating was achieved by heating the mixed solution to 80 °C for 2 h. The as-prepared mixture was centrifuged and washed with ammonia to remove the by-products, and then was centrifuged again and washed with water and alcohol, until no Cl^−^ was detected. The as-obtained sample was dried in a vacuum oven at 80 °C for 12 h, and the as-obtained powders were denoted as Ni-P.

### 2.3. Synthesis of Tunable Crystalline Ni-P

**Figure 1:**
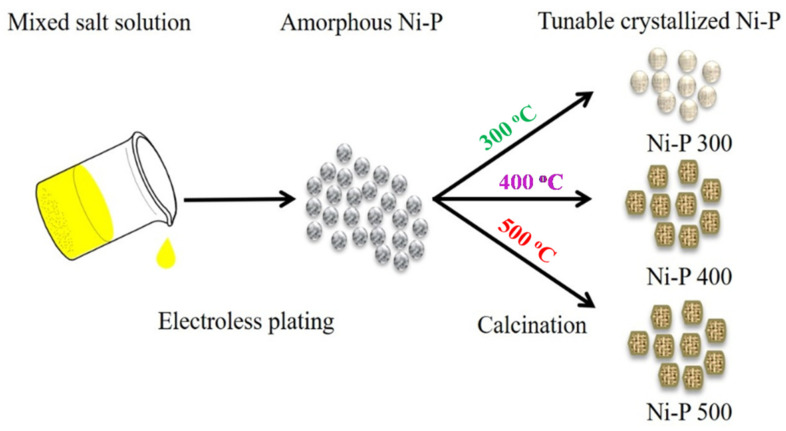
Schematic illustration of synthesis routes of tunable crystallized Ni-P samples.

Based on the reported protocol in the literature [31], the crystalline states of nickel phosphides were tuned, as shown in Figure 1. First, 1 g of the above Ni-P was placed in a reaction vessel and then calcined under a flow of argon in a tube furnace. In order to obtain nickel phosphides with different crystallinities, the calcination temperature was controlled at 300, 400, and 500 °C, respectively. For all samples, the calcination time was 1 h. The as-obtained products at different temperatures are denoted as Ni-P 300, Ni-P 400 and Ni-P 500.

### 2.4. Physical and Chemical Characterization

Scanning electron microscopy (JSM-7100F, JEOL, Pleasanton, CA, USA), transmission electron microscopy (FEI TecnaiF30 F30, FEI Co., Waltham, MA, USA) and energy-dispersive X-ray spectroscopy (EDX) were used to examine surface morphology, microstructures, and element distribution.

The crystallinity and structure phases of the materials were determined by X-ray diffraction (Rigaku RINT2400, Tokoyo, Japan) and Raman (LabRAMHR Evolution) analysis. 

The composition of metal elements was tested by an inductively coupled plasma atomic emission spectrometer (ICP-AES). 

### 2.5. Electrochemical Characterization

All electrochemical tests were carried out on a VMP2 electrochemical workstation (Bio-logic, Pairs, France) using the standard three-electrode system. A Pt foil (Radiometer Analytical, Shanghai, China) and a Cl^−^ free Hg/Hg_2_SO_4_ reference electrode (Radiometer Analytical, Shanghai, China) were used as the counter electrode and the reference electrode, respectively. For the working electrode, 5 mg of catalyst powder was dispersed in 1 mL of isopropyl alcohol, into which 50 μL of Nafion^®^ solution (5 wt%, Dupont, San Mateo, California, CA, USA) was added. The mixture was then homogenized for 1 h in an ice ultrasonic bath to form an ink. An ink aliquot of 20 μL was deposited on a glassy carbon (GC) substrate with a geometric area of 0.5 cm^2^ and dried at 75 °C in a vacuum oven. This catalyst-coated GC was used as a working electrode for the electrochemical measurements. In this work, the Hg/Hg_2_SO_4_ reference was calibrated prior to each measurement in pure H_2_-saturated 0.1 M HClO_4_ solution, using a clean Pt wire as the working electrode. Unless otherwise stated, all potentials recorded using the Hg/Hg_2_SO_4_ reference electrode were converted to potentials with respect to the reversible hydrogen electrode (RHE), according to the following formula: *E*(RHE) = *E*(Hg/Hg_2_SO_4_) + 0.0591 × pH + 0.656. 

The double layer capacitance of the catalysts was recorded in the potential range of 0.2 to 0.4 V, using a range of scan rates from 20 to 120 mV s^−1^ in an Ar-purged 0.1 M HClO_4_ electrolyte. From these data, slopes of the resulting Δ*j*/2 vs. voltage plots (where Δ*j*/2 = |*j*_a_ − *j*_c_|/2, *j*_a_ and *j*_c_ represent the anodic and cathodic current densities at 0.3 V vs. RHE, respectively) were obtained. The polarization curve was measured by linear scanning voltammetry (LSV) in the potential range of 0.1 to −0.4 V, with a scan rate of 5 mV s^−1^. All the obtained results were *IR*-corrected in-situ (85%). Electrochemical impedance spectroscopy (EIS) was performed at an open circuit voltage with an amplitude of 5 mV, in the frequency range of 100 kHz to 0.1 Hz. The stability tests of the catalysts were carried out by chronopotentiometry (CP) at a constant current density of 10 mA cm^−2^. 

## 3. Results and Discussions

**Figure 2:**
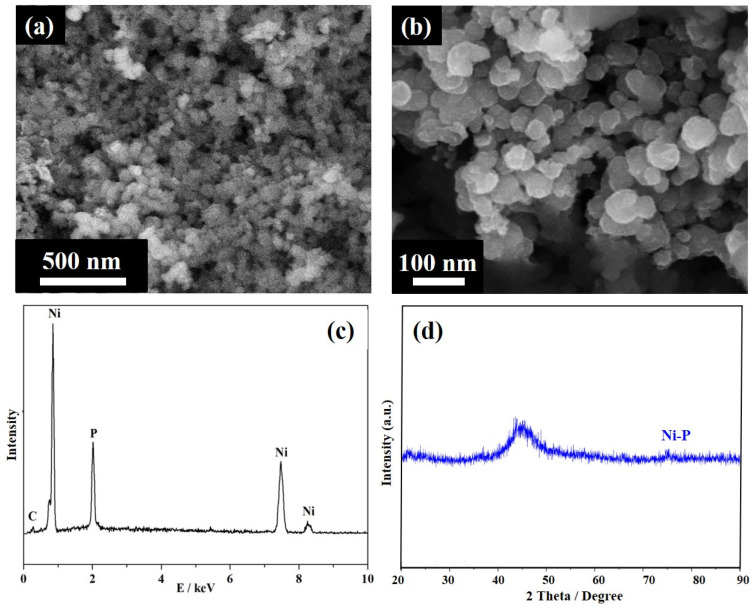
Microstructural characterization of the starting Ni-P catalyst: (**a**,**b**) SEM images with various magnifications, (**c**) EDS spectrum and (**d**) XRD pattern.

Figure 2 shows the morphology and elemental composition of the starting amorphous Ni-P. The SEM images reveal that the as-prepared Ni-P has a spherical morphology, with an average size of nanoparticles in the range of 20 to 30 nm. Figure 2c confirms the presence of the Ni and P elements. The XRD pattern (Figure 2d) of the Ni-P shows very broad diffraction peaks around scattering angles of 44.5, 51.8, and 76.6°, corresponding to the (111), (200), and (220) planes of the face-centered cubic Ni (JCPDS #65–2865) [36]. It can be deduced that the prepared Ni-P shows poor crystallinity and a rather disordered structure. This may result from the presence of P atoms embedded in the Ni lattice, resulting in severe lattice distortion. The grain size of Ni-P nanoparticles, estimated from the Scherrer analysis for the broadening of the (111) peak, was ~1.88 nm. These analyses, i.e., identification of microstructure, elemental composition, as well as the long-range disorder of the sample, confirmed the successful synthesis of highly dispersed amorphous Ni-P.

**Figure 3:**
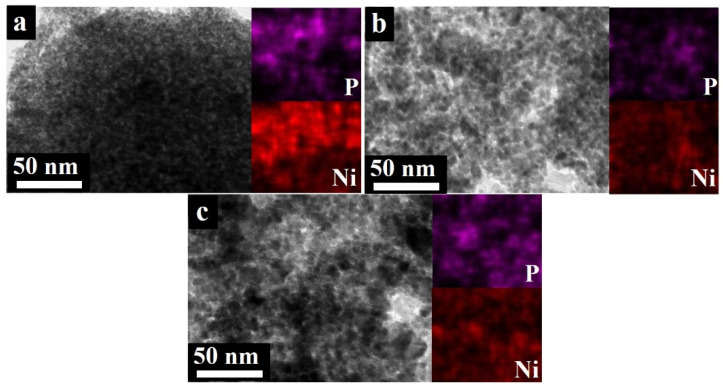
TEM images of the Ni-P crystallized at different temperatures: (**a**) Ni-P 300, (**b**) Ni-P 400, and (**c**) Ni-P 500; and the insets are the EDS mapping images of the corresponding samples.

The amorphous Ni-P was further crystallized via calcination treatment at different temperatures. Both the microstructure and the crystallinity of the as-synthesized samples were further identified. The TEM images of the samples calcined at different temperatures are shown in Figure 3. The TEM images revealed that all the calcined samples consist mainly of fine nanoparticles. In addition, as shown in the EDS elemental mapping (inset in Figure 3), both Ni and P were distributed uniformly in the whole sample. For the Ni-P 300, the spherical structure is maintained, and the nanospheres tend to aggregate into larger nanoparticles, while the nanospheres’ size remains very small (~ 10 nm), with a narrow size distribution. For the Ni-P 400, the nanoparticle size appears to be larger than that of the Ni-P 300, and significantly increases to ~15–20 nm. Unlike the spherical structure of the Ni-P 300, a certain percentage of the Ni-P 400 exhibits uniformly dispersed quasi-faceted morphology. Some studies also reported the same evolution of the morphology, and it can be attributed to the increase in crystallinity [37]. With the further increase in the calcined temperature, the Ni-P 500 sample shows a larger nanoparticle size (around 20–25 nm) and resembles the quasi-faceted morphology. It can be concluded that with the calcination treatment of the amorphous Ni-P, both the nanoparticle size and morphology can be modified. With an increasing calcination temperature, the nickel phosphides tend to grow and exhibit more quasi-faceted morphology. Henkes et al. reported that the calcination temperature could favor the crystallization transformation of amorphous Ni-P and improve the grain growth of nanocrystalline phases [38]. 

**Figure 4:**
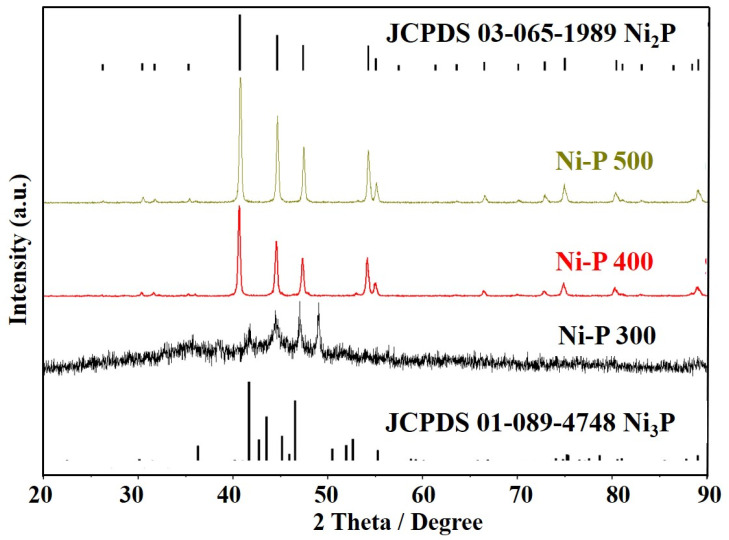
XRD diffraction patterns of Ni-P that crystallized upon different temperatures.

More evidence of the crystallization transformation of nickel phosphides from amorphous phases to nanocrystalline phases after calcination can be observed from the XRD analyses (Figure 4). After the nickel phosphide was calcined at 300 °C, unlike the original amorphous Ni-P, new diffraction peaks appear, which can be ascribed to the crystal planes of the crystallized Ni-P, including 2θ = 41.2, 2θ = 44.6, 2θ = 47.3°, which correspond to the (111), (201) and (210) crystal planes of Ni_2_P (JCPDS #65-1989), respectively, and 2θ = 41.8, 2θ = 48.9°, which correspond to the (231) and (222) crystal planes of Ni_3_P (JCPDS #74-1384), respectively [37,39]. It should be noted that the peaks corresponding to the nanocrystalline Ni_2_P and Ni_3_P phases are still broad, indicating that amorphous Ni-P gradually crystallizes and forms longer range ordering [40]. The calculated grain size for the newly precipitated nanocrystalline of Ni_2_P and Ni_3_P is 12.2 nm and 10.8 nm, respectively. This grain size is in agreement with the TEM observation that shows a mean grain size of about 14.6 nm. The above results show that the Ni-P 300 is a mixture of multiple nickel phosphides, consisting of finely dispersed Ni_2_P and Ni_3_P nanocrystalline. With the increasing calcination temperature, the XRD patterns of the Ni-P 400 and Ni-P 500 show only sharp peaks, which match very well with those of the Ni_2_P crystal structure and no contribution from the Ni_3_P phase is observed. Overall, increasing the calcination temperature results in an increasing crystallinity, and the nanoparticles of the Ni-P 400 and Ni-P 500 contain crystalline Ni_2_P as their major phase. The grain size for the nanoparticles of the Ni-P 400 and Ni-P 500 were calculated to be ~13.8 nm and ~14.6 nm, respectively, indicating a grain growth of nanocrystalline Ni_2_P with the increase in the calcination temperature, which implies that amorphous Ni-P continues to undergo crystallization. First, nanocrystalline Ni_2_P and Ni_3_P phases emerge; then, with a further increase in the temperature, the nanocrystalline Ni_3_P phase disappears. It is speculated that the increase in calcination temperature could favor the grain growth of nanocrystalline Ni_2_P and results in larger nanoparticles, which is consistent with the TEM results. The crystalline phase composition within nanoparticles, nanoparticle size and morphology of the nickel phosphides examined here are closely related to the HER activity of the nickel phosphide catalysts. 

**Figure 5:**
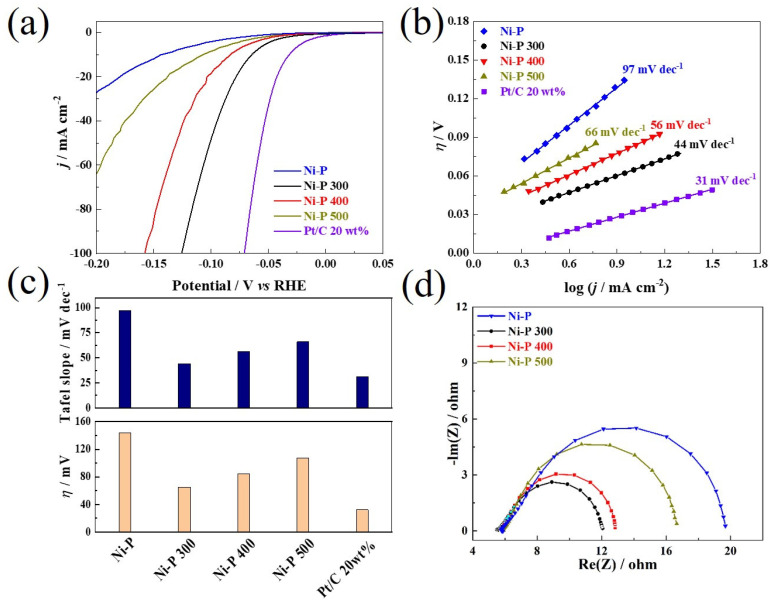
(**a**) *iR*-corrected LSV curves, (**b**) Tafel plots, (**c**) comparison of the Tafel slopes and $\eta_{10}$ and (**d**) the Nyquist plots measured at −0.02 V vs. RHE for the Ni-P and Ni-P that crystallized at different temperatures.

The HER performances of the prepared nickel phosphides with different crystal phases were measured using linear scan voltammograms in 0.1 mol L^−1^ HClO_4_ electrolytes [41]. The mass loading of the nickel phosphides catalysts on all the prepared electrodes was ca. 0.3 mg cm^−2^. All the current density values were normalized to the geometric area of the GC electrode, and the overpotential corresponding to a current density of −10 mA cm^−2^ was defined as $\eta_{10}$. As shown in Figure 5a and Table 1, the amorphous Ni-P shows clear activity towards the HER but requires a large $\eta_{10}$ of ca. 144 mV, which agrees well with the values of $\eta_{10}$ reported for nickel phosphides catalysts in the literature [33]. After the heat treatment of the amorphous Ni-P at different calcination temperatures, the $\eta_{10}$ value of the Ni-P 300 was remarkably reduced to ca. 65 mV, superior to the Ni-P 400 (ca. 84 mV) and Ni-P 500 (ca. 107 mV). Compared with the amorphous Ni-P, the enhanced HER activity of the Ni-P 300 could benefit from the gradual formation of the mixed multiple nickel phosphides, consisting of nanocrystalline Ni_2_P and Ni_3_P. Compared with the Ni-P 300, the decreasing activity of the Ni-P 400 and Ni-P 500 may be ascribed to the increasing crystallinity with the increase in calcination temperature, which could result in the growth of nanocrystalline grains, the coarsening of the surface, as well as the disappearance of the Ni_3_P nanocrystalline phase. By comparison, the HER activity of the Ni-P can be significantly promoted via a facile and controlled calcination treatment (e.g., at 300 °C), which is very close to that of the commercial 20 wt% Pt/C ($\eta_{10}\;$ = ca. 32 mV).

The Tafel slope reflects the reaction mechanism of the HER process, and it is generally used to determine the rate-limiting step of the HER [42]. Figure 5b shows the Tafel curves for the different Ni-P catalysts and the commercial 20 wt% Pt/C. Table 1 summarizes the relevant kinetic parameters obtained from Figure 5b and Figure 5c. Generally, the crystallized Ni-P catalysts exhibit better kinetic parameters than the amorphous Ni-P. The Ni-P 400 and Ni-P 500 show smaller Tafel slopes (ca. 56 mV dec^−1^ and ca. 66 mV dec^−1^, respectively) than that of the Ni-P (ca. 97 mV dec^−1^). It is worth noting that the Ni-P 300 exhibits a very close Tafel slope to that of the 20 wt% Pt/C (Ni-P 300: ca. 44 mV dec^−1^ vs. Pt/C: 31 mV dec^−1^). For both the Ni-P and the crystallized Ni-P catalysts, the Tafel slopes are between 39 mV dec^−1^ and 120 mV dec^−1^, indicating that the HER processes on the catalytic surface are different from that of the Pt-based catalysts, and the HER processes on the Ni-P and the crystallized Ni-P catalysts are in line with the Volmer–Heyrovsky mechanism, where the Volmer process (H^+^ + e^−^ → H_ads_) is the rate-limiting step of the entire HER [43]. Overall, the evolution of the crystallization transformation, as well as morphology of the original amorphous Ni-P via a facile calcination treatment, has a direct impact on the HER activities of the crystallized Ni-P catalysts. Based on the LSV and Tafel analyses, both the $\eta_{10}$, as well as the kinetic parameters of the Tafel slopes, indicate the enhancement of the HER catalytic activity of the Ni-P 300, whereas the Ni-P 400 and Ni-P 500 obtained at higher calcination temperatures show decreased HER activity, mainly related to the increasing crystallinity. The HER kinetics for both the Ni-P and the crystallized Ni-P catalysts are further confirmed by the EIS analyses. Figure 5d displays the Nyquist EIS spectra for the Ni-P catalysts measured at −0.02 V vs. RHE, which corresponded to the hydrogen evolution region. The impendence parameters for the amorphous Ni-P, Ni-P 300, Ni-P 400, and Ni-P 500 catalysts are described in Table 1. These parameters were obtained by fitting the experimental data using ZSimpWin software (AMETEK Inc., New York, NY, USA). The Ni-P 300 shows remarkably improved charge transfer kinetics during the HER compared to the amorphous Ni-P and other crystallized Ni-P catalysts. With the increase in calcination temperature, more Ni and P tend to emerge, along with increased crystallinity. It has been reported that coordinated P could also facilitate the formation of Ni-hydride for subsequent hydrogen evolution via electrochemical desorption and offer more proton-acceptor centers as the active sites and promote the charge transfer of the HER. For the Ni-P 400 and Ni-P 500, the decrease in *R*_ct_ could be related to the increase in the grain size and loss of grain edges due to the fact that single crystalline Ni_2_P is the major phase [33,42]. 

**Figure 6:**
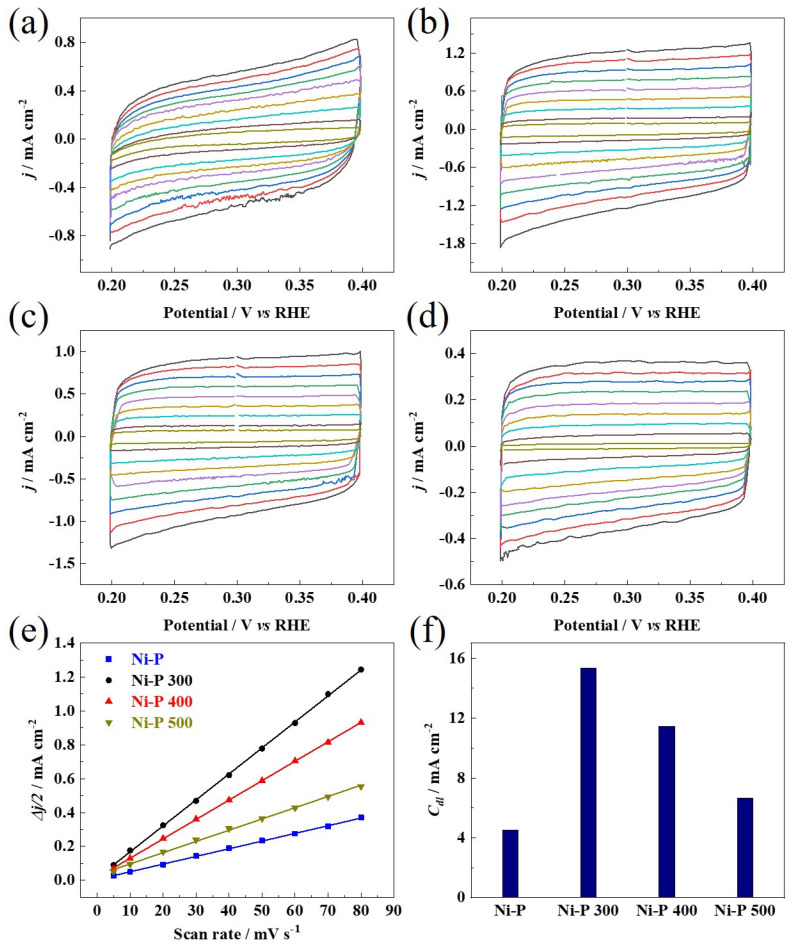
(**a**–**d**) Electrochemical CV curves of the catalysts recorded in non-Faradaic regions at different scan rates of 5 mV s^−1^, 10 mV s^−1^, 20 mV s^−1^, 30 mV s^−1^, 40 mV s^−1^, 50 mV s^−1^, 60 mV s^−1^, 70 mV s^−1^, 80 mV s^−1^, and corresponding to different color lines from inner to outer. (**e**) Plots of the capacitive currents versus the scan rates. (**f**) *C_dl_* value for each catalyst.

The crystallization transformation and the grain growth could also affect the electrochemically active surface area (ECSA) of the Ni-P catalysts, which have previously been reported in the literature [33]. It should be noted that it is still a challenge to precisely estimate the ECSA. Herein, the electrochemical double-layer capacitance (*C_dl_*) of each Ni-P catalyst was determined by integrating the CV curves in the non-faradaic region. It has been reported that the *C_dl_* provides relevant information about the ECSA, which correlates with the number of active sites [44]. As shown in Figure 6 and Table 1, the *C_dl_* values of all the Ni-P catalysts via a calcination treatment show enhancement compared to the amorphous Ni-P, and the increased crystallinity may help in forming more Ni and P configuration, and accordingly create more catalytically active sites. It should be noted that the *C_dl_* of the Ni-P 300 is much higher than those of the Ni-P 400 and Ni-P 500 catalysts, confirming that the mixed nanocrystalline phases of Ni_2_P and Ni_3_P should promote the number of catalytically active sites, due to more defective and strained sites in the Ni-P 300. The lower value of *C_dl_* for the Ni-P 400 and Ni-P 500 could be attributed to the suppressive effect of the further crystallization transformation and the grain growth.

**Figure 7:**
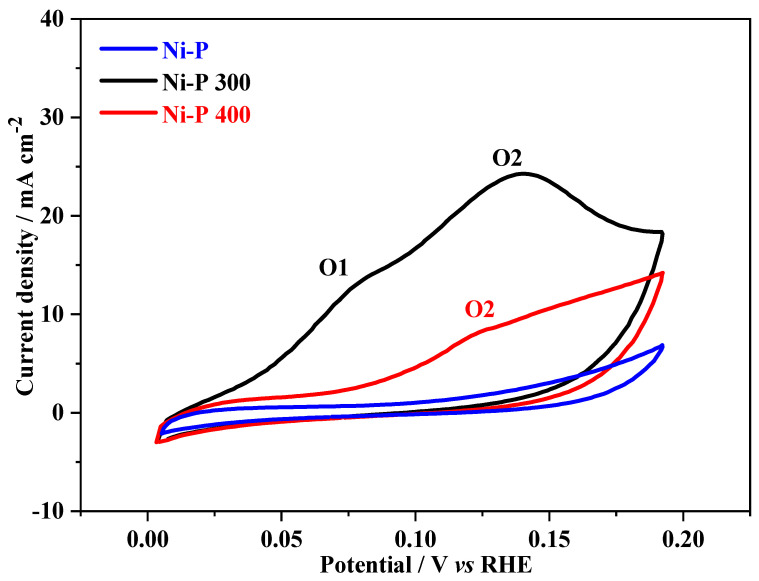
Electrochemical CV curves of the Ni-P catalysts recorded between the potential of 0 and 0.2 V vs. RHE.

Various works have reported that the adsorbed intermediates H_ads_ during the Volmer process are not completely reduced and could remain in the lattice of the HER catalysts [27,29,33]. Thus, oxidation peaks of H_ads_ can be observed in the oxidation process of forward-sweep [42,44]. Prior to the forward-sweep LSV test, the electrodes with different Ni-P catalysts initially underwent HER processes at 20 mA cm^−2^ for 1 h. Figure 7 presents the LSV curves between the potential of 0 and 0.2 V vs. RHE. There are clear oxidation peaks of H_ads_ for the Ni-P 300 and Ni-P 400, but no oxidation peaks can be observed for the amorphous Ni-P. These oxidation peaks are believed to be related to the H_ads_, which does not desorb in time, and the remaining adsorption state in the lattice of Ni-P catalysts. Interestingly, there is an oxidation peak (O2: 0.13–0.16 V) for both Ni-P 300 and Ni-P 400. However, one more oxidation peak (O1: 0.13–0.16 V) can be observed for Ni-P 300. This indicates that there is another state of H_ads_ in the crystal lattice. Considering the presence of distinct crystallographic phases in the Ni-P 300 and Ni-P 400, we speculate that the oxidation peak O2 and the oxidation peak O1 can be attributed to the Ni_2_P and Ni_3_P phases, respectively. Therefore, it can be considered that for the Ni-P 300 with mixed nanocrystalline phases, it has a combined ability to adsorb active hydrogen, that is, both the Ni_2_P and Ni_3_P nanocrystalline phases will contribute to the HER process and provide active sites. The surface charges (Q_s_) that are derived from the integration of the forward-sweep LSV curve can be used to evaluate the adsorption/desorption abilities of the Ni-P catalysts. The Q_s_ values of the Ni-P 300 are much higher than the amorphous Ni-P, as well as for Ni-P 400, indicating that the Ni_2_P and Ni_3_P nanocrystalline in the Ni-P 300 helps provide more activity sites or/and decreases the reaction activation energy of the adsorption/desorption process, thus promoting the reactivity.

**Figure 8:**
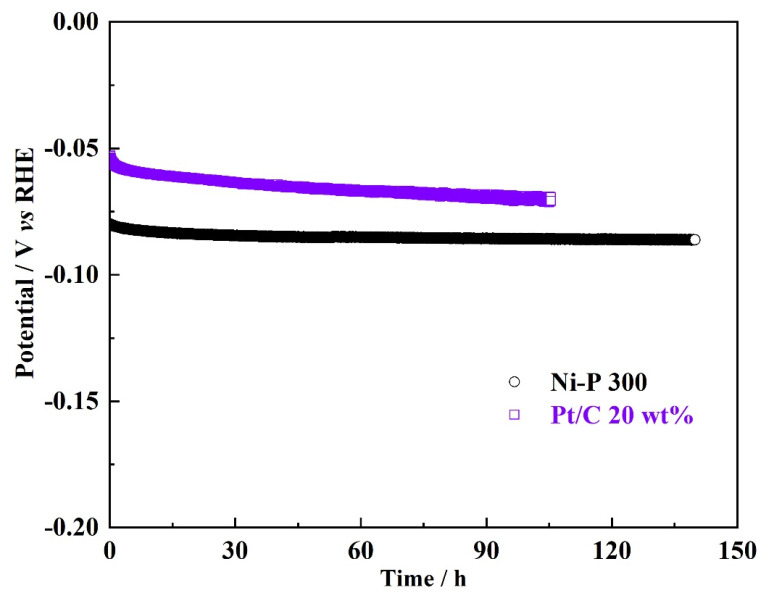
Chronopotentiometric curves of the Ni-P 300 and commercial 20 wt% Pt/C recorded at a constant current density of 10 mA cm^−2^.

Stability is a key challenge for electrocatalysts to meet the demanding targets of practical applications in water electrolyzers. The electrochemical stability of each catalyst was evaluated by the chronopotentiometric (CP) method at a constant current density of 10 mA cm^−2^ in 0.5 M H_2_SO_4_ electrolytes [28]. Figure 8 shows that the Ni-P 300 has negligible degradation during 140 h of galvanostatic electrolysis, and the potential required to achieve the current densities of 10 mA cm^−2^ increases only from −79 mV to −85 mV. The slight increase in potential may be a result of the bubble effect or the slow dissolution, in addition to the physical detachment of the catalysts during the constant hydrogen evolution process. In contrast, the commercial Pt/C exhibits worse catalytic stability than that of the Ni-P 300. The potential required to achieve the current density of 10 mA cm^−2^ increases significantly from −53 mV to −70 mV during the 105 h galvanostatic electrolysis. There are many literature reports on the degradation mechanism of commercial Pt/C catalysts during the HER that attribute the decay in the stability to the migration and detachment of Pt NPs from the carbon support, when a cathodic overpotential is applied. Despite slightly lower catalytic activity of the Ni-P 300, compared with the commercial Pt/C catalyst, all the above results can verify that the Ni-P 300 shows superior stability to the commercial 20 wt% Pt/C. We propose that the geometry optimization, as well as the use of support, would further improve the catalytic activity of the Ni-P 300, which makes the Ni-P an advanced type of non-precious HER catalyst for water electrolysis. Further development of this type of catalyst could have important consequences for PEMWE technology and production of green hydrogen. 

## 4. Conclusions

In this work, nickel phosphides with increased crystallinity and tunable properties were prepared from amorphous Ni-P at different calcining temperatures. The impact of crystallographic phases and their crystallinity on the HER activity and stability were systematically explored. The structure and composition of the crystallized Ni-P catalysts were characterized by SEM, TEM, EDS, and XRD. The results show that Ni-P 300 is a mixture of multiple nickel phosphides, consisting of finely dispersed Ni_2_P and Ni_3_P nanocrystallines, while Ni-P 400 and Ni-P 500 contain crystalline Ni_2_P as their major phase, in addition to grain growth. On average, the Ni-P 300 catalyst shows comparable HER performance (the overpotential is only ca. 65 mV at a current density of −10 mA cm^−2^) and better stability during 140 h of galvanostatic electrolysis than that of the commercial 20 wt% Pt/C. In summary, the crystallized Ni-P catalysts exhibit better kinetic parameters than that of the amorphous Ni-P and could provide more activity sites, promote the H_ads_ adsorption/desorption abilities, and thus improve the HER performance.

## Figures and Tables

**Table 1:** Summary of electrochemical parameters of different catalysts.

	$\eta_{10\;}{}_{mA\;cm^{-2}}$ (mV vs. RHE)	Tafel Slope (mV dec^−1^)	*R_ct_* (Ω cm^−2^)	*C_dl_* (mF cm^−2^)
Ni-P	144	97	14.1	4.5
Ni-P 300	65	53	6.3	15.3
Ni-P 400	84	56	6.8	11.5
Ni-P 500	107	66	10.9	6.6
Pt/C 20 wt%	32	31	/	/

## Data Availability

Data will be available upon request from the corresponding authors.
